# Variation of C peptide decay rate in diabetic patients with positive glutamic acid decarboxylase antibody: better discrimination with initial fasting C peptide

**DOI:** 10.1186/1472-6823-13-10

**Published:** 2013-03-01

**Authors:** Xia Li, Gan Huang, Jian Lin, Lin Yang, Zhiguang Zhou

**Affiliations:** 1 Key Laboratory of Diabetes Immunology, Ministry of Education; Diabetes Center, Metabolic Syndrome Research Center, Institute of Metabolism and Endocrinology, 2nd Xiangya Hospital, Central South University, No.139 Middle Renmin Road, 410011, Changsha, Hunan, P.R. China

## Abstract

**Background:**

Diabetic patients with positive glutamic acid decarboxylase antibody (GAD-Ab) could be classified as autoimmune diabetes, which is discriminated into acute-onset classical type 1 diabetes (T1DM) and latent autoimmune diabetes in adults (LADA). However, whether the decay rate of beta cell function is relevant with the mode of onset (acute or latent-onset) is unclear. We aimed to investigate whether initial C peptide levels could help differentiate variation of C peptide decay rate.

**Methods:**

Five hundred and twenty-seven newly diagnosed GAD-Ab positive diabetic patients were followed up to assess the natural course of beta cell function. Beta cell function failure was defined as fasting C peptide and postprandial C peptide levels less than 100 pmol/L and 150 pmol/L respectively.

**Results:**

All these diabetic patients were discriminated according to initial fasting C peptide of 300 pmol/L, that is B+ (larger than 300 pmol/L) and B- (less than 300 pmol/L) group. The proportion of developing beta cell function failure was 13.1% in B+ group and 90.5% in B- group, which suggested that fasting C peptide levels made a good distinction of the heterogeneity in autoimmune diabetes. Receiver operator characteristic (ROC) analysis suggested that the fasting C peptide level of 300 pmol/L was optimal for determining beta cell function failure with sensitivity of 90.5% and specificity of 86.9%.

**Conclusions:**

Initial level of fasting C peptide is a good indicator for predicting beta cell function failure in GAD-Ab positive diabetic patients.

## Background

The WHO recommendation in 1999 on classification of diabetes is now based more and more on the etiological evidence, with autoimmunity being the most direct and measurable evidence ever since the standardized radioligand assays for islet autoantibodies have been esta blished [1]. So, more and more clinically diagnosed “type 2” diabetes has been re-classified as latent autoimmune diabetes in adults (LADA), a subtype of type 1 diabetes [2,3]. However, except for the etiology of diabetes, clinicians are paying more attention on the progression of beta cell function. Evidence from UKPDS, DCCT, and other studies has demonstrated that beta cell function decline is closely related with the all-sides glycemic control and the diabetic complications [4]. So, to make a distinction between the different destruction speed of beta cell function in autoimmune diabetes is important and LADA is specially proposed as a subtype for this reason. According to the diagnostic definition proposed by the Immunology of Diabetes Society (IDS), LADA is specially referred to patients over 30 years of age, positive for at least one islet autoantibodies, and not treated with insulin within the first 6 months after diagnosis [5]. So, the age (adult, over 30 years) and mode (latent, no need for insulin at the initial stage) of onset are regarded as discriminative factors for autoimmune diabetes. However, “latent” does not always “adult-onset”, and patients with “latent autoimmune diabetes in the young (LADY)” was also put forward because of the relative slow progression [6].

Glutamic acid decarboxylase antibody (GAD-Ab) is the most widely used and the most valuable autoimmune markers in diagnosing autoimmune diabetes, especially for Chinese and adult-onset patients. [7,8]. Previous studies reported that GAD-Ab titer could help identify the heterogeneity of LADA patients with LADA-type 1 and LADA-type 2 diabetes [9,10]. But for juvenile- and adult-onset autoimmune diabetes, the status and the titer of GAD-Ab is not so helpful [11]. So, to address possible predictors of the discrepancy of beta cell loss, we have conducted a prospective study to characterize the difference of 527 cases of autoimmune diabetes, who have been followed closely and assessed serum C peptide levels repeatedly. Our results indicated that initial beta cell function was the most usefulmarker for differentiating beta cell failure tendency in autoimmune diabetes.

## Methods

### Inclusion of subjects and data collection

This study was carried out according to the Helsinki guidelines. All patients gave informed consent to participation in the study and the protocol was approved by the Research Ethics Committee in Second Xiangya Hospital, Central South University. A total of 4,469 newly diagnosed patients who were hospitalized from July 2002 to July 2006 were screened for GAD-Ab; five hundred and twenty-seven patients with positive GAD-Ab were invited and agreed to participate in this study as they fulfilled the following criteria: (1) diabetes diagnosed according to the report of WHO in 1999; (2) disease duration less than one year; (3) GAD-Ab positive testing twice within one month. The disease onset was defined as the initial diagnosis of diabetes and disease duration was thus calculated.

All patients were treated according to the decision of individual physician and insulin or metformin were applied in accordance with the seriousness of the case and adjusted in line with blood glucose levels,other kinds of hypoglycemic agents including sulfonylureas and thiazolidinediones were not employed. Data from all patients, including age of onset, diabetes duration, body mass index (BMI), ketosis were collected. Serum was collected for measurement of HbA1c, islet autoantibodies and C peptide levels.

### Ethics

We have complied with the recommendations of the Declaration of Helsinki. This study was approved by the ethics committee of Second Xiangya Hospital, Central South University and informed consent was obtained from all participants.

### Definition of clinical subtype of autoimmune diabetes

All these autoimmune diabetes were subdivided into two subtypes, that is the acute-onset T1DM and latent-onset LADA. Acute-onset autoimmune T1DM was defined by 1) Requirement of insulin therapy since diagnosis; + 2) positive GAD-Ab. Latent-onset LADA was defined by 1) No ketosis or ketoacidosis during the first 6 months after diagnosis; + 2) no requirement of insulin therapy during the first 6 months after diagnosis; + 3) positive GAD-Ab.

### Diabetes associated autoantibody analysis

Serum was analyzed for the presence of GAD-Ab by highly sensitive and specific quantitative radioligand binding assay methods as reported previously [12]. Intra-assay and inter-assay coefficients of variation were 8.9% and 11.2% (n=5) respectively. The cut-off point of GAD-Ab index was determined according to the 99.5% upper limit of 188 healthy Chinese controls. The GAD-Ab index of 0.05 or higher was defined as positive.

### Human leukocyte antigen (HLA-DQ) genotyping

DNA was isolated from peripheral blood leukocytes using the phenol–chloroform method [13]. The polymorphic second exons of DQA1, DQB1 were amplified using PCR. The HLA-DQA1 and -DQB1 types were defined by DNA analysis using polymerase chain reaction (sequencing-based typing). The frequencies of HLA-DQA1 and –DQB1 genes were tested; the susceptible and protective haplotypes were defined according to our previous report and HLA-DQA1*03-DQB1*0303, DQA1*05-DQB1*0201 and DQA1*03-DQB1*0401 are susceptible to type 1 diabetes, and HLA- DQA1*0102-DQB1*0602 is protective haplotypes [7].

### Measurement of beta cell function

Beta cell function was assessed in all patients in two ways: 1) fasting plasma C-peptide (FCP) concentration; 2) postprandial C-peptide (PCP): Sera were determined 2 h after a mixed meal (with about 400 cal). C-peptide levels were measured at the time of initial inclusion (after glucotoxicity had been removed) and during the follow-up.

Beta cell function failure was defined as FCP and PCP levels less than 100 pmol/L and 150 pmol/L respectively, and the initial beta cell function was defined as preserved (B+) if FCP level was higher than 300 pmol/L or PCP level higher than 600 pmol/L, and those whose C peptide levels could not meet the above criteria were defined as B-. These cutoff values are determined according to the results previously reported to differentiate normal subjects, type 1 and type 2 diabetic patients [14].

### Statistical methods

The statistical analysis was done with SPSS 16.0 software. Data were shown by means ± SD or as indicated. ANOVA was used to assess differences in continuous variables (age, age at diagnosis, HbA1c on admission, BMI, HbA1c, C-peptide levels). When this test indicated that there were significant differences between groups, pair-wise comparisons were made to identify those differences. Chi-square test was used in comparison of classification data. All significance tests were performed as P-value less than 0.05 and were two-tailed.

## Results

### Patient characteristics and different tendency of beta cell function

Among all the 527 cases of autoimmune diabetes, the mean age was 39.1±19.1 yr, 54.6% were males, with the mean duration of 8.3±1.7 months,and all patients were newly onset diabetes with duration less than one year. One hundred and seventy-eight cases were defined as beta cell function failure (FCP and PCP levels less than 100 pmol/L and 150 pmol/L respectively) at the beginning of the study and thus were not followed up. Forty-nine patients descended to beta cell failure when followed up for 12 months and 24 failure patients at 24 months, 14 at 36 months, 17 at 48 months. Altogether, 104 patients developed beta cell function failure during the 48 months’ follow up.

### Difference of beta cell function failure in acute-and latent-onset autoimmune diabetic patients

We tested whether patients in T1DM and LADA had disparate progression of beta cell function. Among all the 258 T1DM cases, 216 patients developed beta cell failure at the onset or during follow-up of the study, with the proportion of 83.7%; while for LADA patients, 24.5% developed beta cell failure at the onset or during follow-up of the study.

### Different clinical features in patients with distinct variation of beta cell function

All the autoimmune diabetic patients were divided into three subgroups according to their distinct outcomes of beta cell function, patients with beta cell function failure at the onset of the disease (group 1); patients who developed beta cell function failure during the follow-up (group 2); patients who did not develop beta cell function failure during the follow-up (group 3). The clinical features of three subgroups were listed in Table 1. Patients in group 1 had the highest frequency of HLA-DQ susceptible haplotypes, while the most frequency of protective haplotypes was found in group 3, which suggested the HLA-DQ gene susceptibility present a continuous spectrum in patients with different beta cell function. Compared with patients who had beta cell function failure (group 1 + group 2), those who did not develop beta cell function failure had later age of onset, less frequency of kenosis-prone diabetes, lower titer of GAD-Ab, and higher FCP levels. The clinical features of those who had beta cell failure at the onset stage and patients who developed beta cell function failure later were similar in onset age, ketosis tendency, HbA1c level, however, the serum FCP levels were significantly different in two groups.

**Table 1:** Clinical features in patients with distinct variation of beta cell function

	**Group 1: Patients with beta cell function failure at the onset of the disease (n=178)**	**Group 2: Patients who developed beta cell function failure during the follow-up (n=104)**	**Group 3: Patients who did not develop beta cell function failure during the follow-up (n=245)**
Sex (Male%)	54.5%	54.8%	54.7%
Age of onset (yr)	25.4±15.4	29.3±15.8	46.4±14.9*
Ketosis (%)	81.5%	68.3%	17.1%*
HbA1c	11.0±3.4	10.7±3.5	9.3±3.3
FCP (pmol/L)	25.2(0–99.0)	202.3(102.3–1544.0)#	526.6(114.3–3172.0)*
PCP (pmol/L)	56.9(0–3851.7)	452.3(80.4–5976.4)#	1304.0(161.0–2728.1)*
GAD-Ab	0.52(0.05–2.0)	0.48(0.05–2.34)	0.14(0.05–2.28)*
Percentage of T1DM	81.5%	68.3%#	17.1%*
Percentage of LADA	18.5%	31.7%#	82.9%*
HLA-DQ susceptible haplotypes	105/178 (59.0%)	44/104 (42.3%)	86/245 (35.1%)*
HLA-DQ protective haplotypes	8/178 (4.5%)	6/104 (5.8%)	21/245 (8.6%)*

Note: Compared with patients who had beta cell function failure (group 1 + group 2), patients in group 3 who did not develop beta cell function failure had later age of onset, less frequency of kenosis-prone diabetes, lower titer of GAD-Ab, and higher FCP levels. When compared with group 1 and group 2, * P<0.05. The clinical features of group 1 and group 2 were similar in onset age, ketosis tendency, HbA1c level, however, the serum FCP levels were significantly different in two groups. When compared with group 1, #P<0.05.k.

### Factors which influence the decline of beta cell function

To evaluate the possible risk factors contributing to the development of beta cell failure, multivariate analysis and a Cox regression analysis was performed (age of onset, diabetes duration, frequency of ketosis, BMI, initial fasting C peptide, GAD-Ab titer, HLA-DQ haplotypes were analyzed as inclusive factors). Multivariate analysis showed that age of onset, the frequency of ketosis, BMI and FCP levels were significantly correlated with distinct outcomes of beta cell function. Subsequently, the results of Cox regression analysis revealed that influencing factor included FCP levels, age of onset and BMI, with the largest coefficient of determination (0.89) for initial FCP levels.

**Figure 1:**
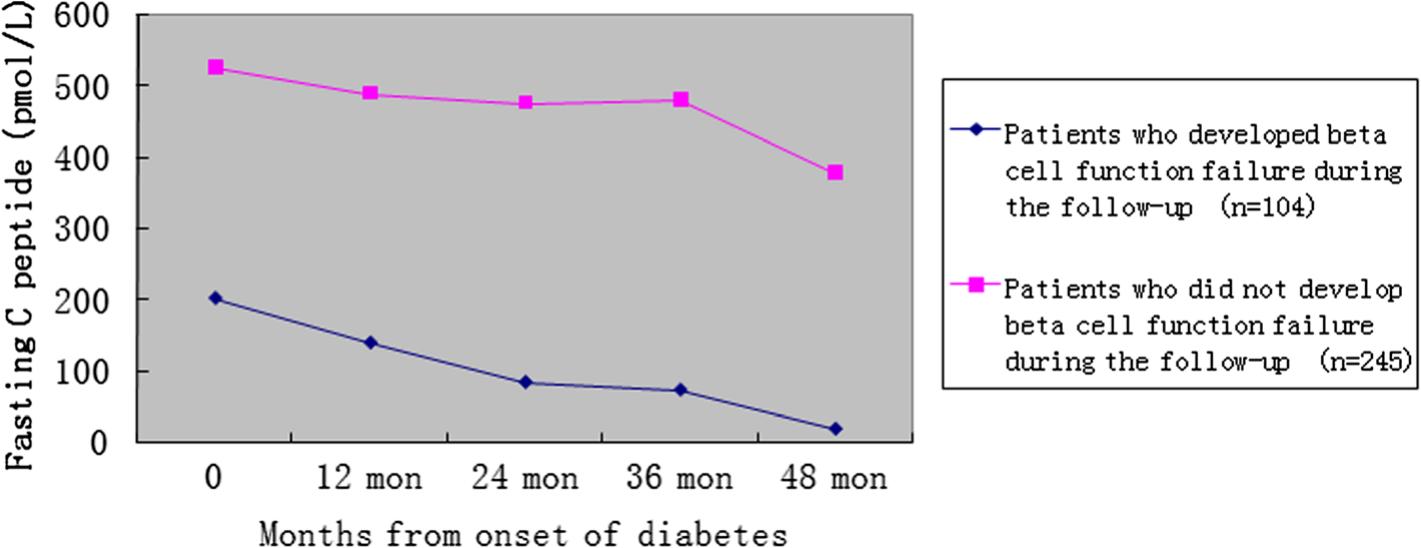
**Changes of fasting C peptide in different autoimmune diabetic patients with distinct endings of beta cell function.** Changes of fasting C peptide (FCP) in different autoimmune diabetic patients with distinct endings of beta cell function were shown, and FCP made a dramatic discrimination in the C peptide decay rate.

The natural tendency of C peptide levels during the follow-up was shown in Figure 1. The fasting C peptide levels in other two groups with different ending of beta cell function were dramatically different (178 patients with beta cell failure were not followed up). The above results suggested that initial FCP levels were closely related with the prognosis of beta cell function in autoimmune diabetes.

### Re-classification of autoimmune diabetes with initial beta cell function

The above results suggested that initial FCP levels maybe the promising predictor of possible beta cell function failure. We arbitrarily defined better preservation of beta cell function (B+) when the FCP levels were more than 300 pmol/L and worse beta cell function (B-) when FCP were less than 300 pmol/L. Follow-up study showed that a dramatic difference of beta cell function was found, 13.1% in B+ group while 90.5% in B- group progressed to beta cell function failure. Considering the proportion of 83.7% in T1DM and 24.5% in LADA progressed to beta cell failure, the results showed that initial serum FCP level made a better distinction of the heterogeneity in autoimmune diabetes than WHO recommendation with LADA sub-typing.

**Figure 2:**
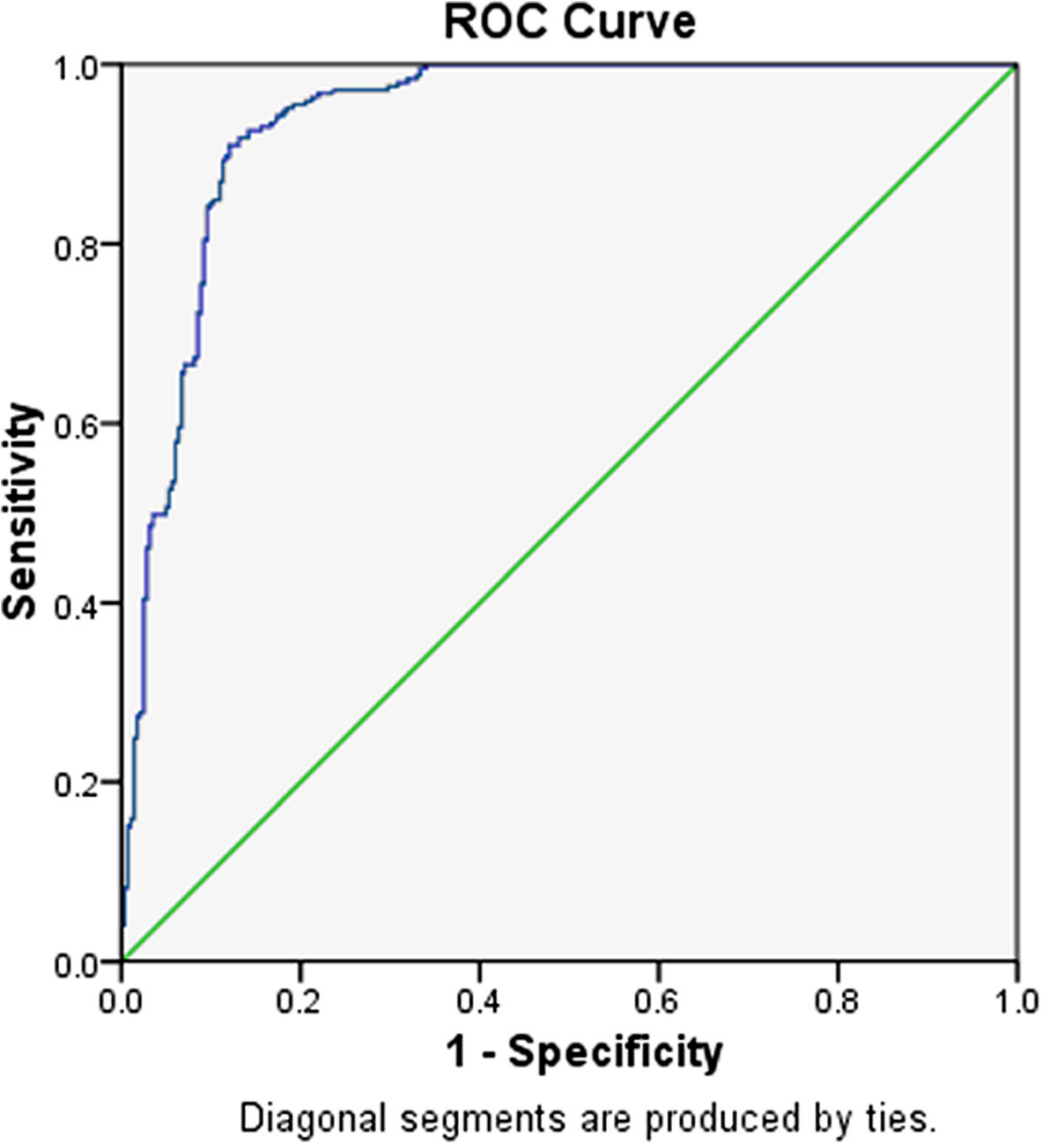
**Receiver operating curve analysis of fasting C peptide in differentiating beta cell function in autoimmune diabetes.** The differentiating diagnostic value of fasting C peptide (FCP) in autoimmune diabetes was shown in the receiver operating curve (ROC). The top left corner of the curve represented the optimal FCP cur-off point, which was at the level of 300 pmol/L.

We then tested the optimal FCP levels in defining better or worse beta cell function preservation. ROC analysis found that the FCP level of 300 pmol/L was optimal (Figure 2), with sensitivity of 90.5% and specificity of 86.9% for determining beta cell function failure. The sensitivity and specificity of FCP level of 200 pmol/L and 400 pmol/L were 96.3%, 80.7%, 78.6% and 87.9% respectively.

## Discussion

Islet beta cell function plays an important role in the pathogenesis of diabetes mellitus, and the destiny of beta cell function failure leads to the uncontrollable hyperglycemia in long term and diabetic complications. From the patients’ point of view, it is of highest priority to find factors for the discrimination of beta cell function course. For autoimmune diabetes, beta cell function failure is more apparent as the immunity mediated beta cell destruction [15]. However, the heterogeneous tempo of beta cell function failure in autoimmune diabetes was recognized and LADA is subdivided specially [16,17]. However, the arbitrary classification scheme using onset age to categorize the heterogeneity of autoimmune diabetes has some limitations; 1) the confirmation of the exact onset age is not so certain, 2) the onset age is a continuous parameter, the optimal cut off point is undecided [18], 3) the term “latent” in LADA could not be reflected by the “late onset” [19], classical type 1 diabetes in adults is common, and “young onset” could also be “latent”.

So, we aimed to investigate whether initial C peptide levels is useful marker for differentiating variation of C peptide decay rate. There were three different outcomes of beta cell function progression, that is, beta cell function failure at the onset of the disease, failure during the follow-up and no failure during the follow-up, and initial fasting C peptide levels was dramatically different in patients with distinct progressive endings of beta cell function. So, we proposed that the disparate outcomes of beta cell function in autoimmune diabetes could be better discriminated by initial FCP levels. When defined beta cell function as B+ or B- by FCP level of 300 pmol/L, we found 13.1% in B+ group while 90.5% in B- group progressed to beta cell function failure. So, initial FCP levels made a better distinction of the heterogeneity in autoimmune diabetes with the sensitivity of 90.5% and specificity of 86.9%.

Positive islet autoantibodies in autoimmune diabetes patients are thought to indicate a progressive autoimmune disease in the beta cells associated with a gradual decrease in insulin secretion. So, islet autoantibodies (A) was applied as one marker to dissect the heterogeneity of ketosis prone diabetes by Maldonado et al., beta cell function (B) was also used, and four different combination of AB groups with diverse clinical features were found. Therefore, the classification scheme using A (autoantibody) and B (beta cell function) was proposed in discriminating ketosis-prone diabetes [20].

For antibody positive autoimmune diabetes, it is likely that the beta cell destruction continues after diagnosis, with varying rate until the beta cells are depleted [21,22]. C-peptide levels at diagnosis, age, gender, titer of GAD-Ab, degree of obesity and puberty, and levels of HbA1c are factors reported to influence beta cell function after diagnosis [23]. In our study, we found that initial beta cell function reflected by fasting C peptide levels might be a good marker for dissection the heterogeneity of autoimmune diabetes. The possible reasons are, 1) the value of FCP levels had a higher sensitivity and specificity in predicting beta cell failure; 2) initial C peptide levels could be a mixed reflection of age onset, predisposing genes, the extent of insulin resistance and insulin deficiency. Consistently, Thunander M et al. followed LADA patients for three years, and determined beta-cell function with early insulin vs conventional treatment, and found only baseline level of C-peptide significantly influenced C-peptide level after 3 years [24]. Other study reported that initial C-peptide is a predicting factor for effectiveness of insulin intervention for LADA [25]. For GAD-Ab positive LADA patients (without classical type 1 diabetes), the same trend of C-peptide declining curve in patients with different initial C-peptide levels, as shown in our Figure 1, had been also reported [26]. So, the use of initial beta cell function may have important implications in predicting the prognosis of autoimmune diabetes.

## Conclusions

Based on this study, we conclude that initial level of fasting C peptide, instead of age and mode of onset, is a better criterion for predicting beta cell function failure in GAD-Ab positive autoimmune diabetes.

## Competing interests

All authors declare that they have no competing interests.

## Authors’ contributions

XL: conception and design of the study; analysis and interpretation of data; drafting the manuscript; GH, JL and LY: conception and design of the study; acquisition data; ZZ: conception and design of the study; and interpretation of data; revising the manuscript. All authors critically revised the manuscript for important intellectual content and approved the final version of the manuscript.

## Pre-publication history

The pre-publication history for this paper can be accessed here:

http://www.biomedcentral.com/1472-6823/13/10/prepub
